# The Dull or Backward Child

**Published:** 1907-02-09

**Authors:** 


### The Dull or Backward Child.

When rival theologians have at last settled what religious dogmas are to be taught to children, when public opinion has realised that education is concerned not only with the mind or even the soul, but also with the body, and when, in consequence, medical inspection of school children has become a routine practice, then, and not until then, the dull or backward child will come by his own, and will receive the attention which he deserves; this in most cases he will fully repay. At present, such a child is forced to attend a school where he is liable, either to be altogether neglected, or to have his life rendered a burden to him, and where he exhausts alike the time and the patience of his teachers and is a drag on the progress of his more intelligent fellow-scholars. Yet all these evils may be, and in fact usually are, the results of some readily remediable cause.

First of all, however, it is important to distinguish between the child who is merely dull or backward, and the one who has definite mental deficiency, for parents are usually extremely loath to admit even a suspicion of imbecility in their children, and sometimes speak of those who are obviously not far removed from idiocy as simply "backward." In the words of Dr. Charles West: "A mentally deficient child would be abnormal for any age, whereas a backward child is merely abnormal for its own age." The mentally deficient child has some definite brain lesion, sometimes gross, sometimes incapable of clinical definition, and his condition is essentially incurable, although education on special lines may enable him to become "a hewer of wood and a drawer of water." Mere dullness, on the other hand, is due usually to some disorder of other parts of the body or of the general nutrition, with, as a result, an imperfectly nourished brain.

To what causes, then, may dullness be due? First of all, to under-feeding, the deficiency being perhaps quite as often in the quality as in the quantity of the food. It is true that starvation acting on a nervous temperament may produce a sort of acute precocious cleverness; but in the long run, the nutrition of the brain in such circumstances must suffer, and so its receptive powers be diminished. It is the merest truism to say that it is useless to try to teach a half-starved child. This is far from being a question simply of the poverty or vice of the parents. If the art of cooking, the art of making a tasty, and at the same time nourishing, meal out of apparently unpromising materials, were only half as well understood in this country as it is in France, a vast amount of under-feeding would at once disappear. Then there are always a number of children attending school who are suffering from definite chronic illness—children with heart-disease—congenital or acquired—anæmia, tuberculous disease in its early stages, and so on. The parents may be profoundly ignorant, or perhaps utterly careless, the teachers often unable to distinguish between moral perversity or sheer idleness and the apathy of physical weakness or disease, and so these unfortunate children may drag wearily on at school for a long time before the true cause of their mental condition is discovered.

Yet another, and often quite unsuspected, reason for mental dullness is slight chorea. In the majority of cases the characteristic movements are at first but slight, and therefore easy to interpret wrongly; and there are some patients in whom, throughout the disease, the movements are comparatively unobtrusive. Such children are apt to get into trouble, not only for making grimaces and for dropping things, but also for inattention or indifference at their lessons. Marked jerky movements are apt to be regarded by non-medical observers as the essential feature of chorea, whereas, as a matter of fact, loss of muscular power and mental weakness are always present to some extent, and either one or the other may be the predominating symptom. In some instances an altered mental condition may be a very prominent and a very misleading symptom, and even in the mildest cases of chorea the child is unable to concentrate his attention on any subject for more than a very short time.

Finally, and in many respects most important of all as a cause of chronic dullness, are adenoid growths in the naso-pharynx. They are well known to lead often to deafness, and this necessarily makes a child appear dull and stupid; but, in addition, they interfere with the air entry into the lungs, and so qualify the aëration of the blood; consequently the child's brain is supplied with blood which is poorly oxygenated, and hence its functions are imperfectly performed. Hundreds, perhaps thousands, of school children in this country suffer in this way, and yet the real cause of their slow progress, both mental and physical, is never suspected until possibly irremediable mischief has been done. Now all these causes of mental dullness could be detected, and most of them completely removed, under efficient medical inspection. Can there be any stronger argument to indicate the urgent need for the speedy enforcement of this measure, with its almost untold possibilities of benefit to the rising generation?

